# Impact of off-center diagonal profile depth pairing on gamma pass rates in portal dosimetry

**DOI:** 10.1093/jrr/rraf071

**Published:** 2025-11-24

**Authors:** Yuya Suzuki, Atsushi Yamashita, Yoshiaki Okada, Takuya Ochiai, Kouyou Ishida, Kenji Ota, Toshio Ohashi

**Affiliations:** Department of Radiological Technology, Tokyo Saiseikai Central Hospital, 1-4-17 Mita, Minato-ku, Tokyo 108-0073, Japan; Department of Radiological Technology, Tokyo Saiseikai Central Hospital, 1-4-17 Mita, Minato-ku, Tokyo 108-0073, Japan; Department of Radiological Technology, Tokyo Saiseikai Central Hospital, 1-4-17 Mita, Minato-ku, Tokyo 108-0073, Japan; Department of Radiological Technology, Tokyo Saiseikai Central Hospital, 1-4-17 Mita, Minato-ku, Tokyo 108-0073, Japan; Department of Radiological Technology, Tokyo Saiseikai Central Hospital, 1-4-17 Mita, Minato-ku, Tokyo 108-0073, Japan; Department of Radiological Technology, Tokyo Saiseikai Central Hospital, 1-4-17 Mita, Minato-ku, Tokyo 108-0073, Japan; Department of Radiation Oncology, Tokyo Saiseikai Central Hospital, 1-4-17 Mita, Minato-ku, Tokyo 108-0073, Japan

**Keywords:** EPID, portal dosimetry, fluence, off-center diagonal profile, patient-specific QA

## Abstract

This study evaluated the impact of off-center diagonal (OCD) profile depth pairing between the treatment planning system (TPS) and the electronic portal imaging device (EPID) on gamma pass rates in portal dosimetry. In clinical workflows, OCD profiles are used in the TPS to generate predicted images via the portal dosimetry image prediction (PDIP) algorithm and in the EPID system to correct measured fluence. The consistency of these settings may influence verification accuracy. Portal images were acquired using a TrueBeam linear accelerator with an aS1200 EPID for four photon energies: 6X, 10X, 6 flattening filter-free (FFF) and 10FFF. Five OCD profiles (reference depth, 5, 10, 20 and 30 cm) were configured in both the PDIP model and EPID system. For each energy, a total of 175 plan–measurement combinations were evaluated, derived from five PDIP OCD depths combined with five EPID OCD depths across seven field sizes. Field sizes ranged from 5 × 5 to 30 × 30 cm^2^. Gamma analysis used 3%/3 mm criteria with a 10% dose threshold. A two-way analysis of variance assessed the effects of TPS and EPID OCD depths and their interaction. For 6X and 10X beams, pass rates varied with configuration, showing better agreement when depths were matched or EPID was deeper. In contrast, 6FFF and 10FFF beams maintained high pass rates with minimal variation. These findings indicate that OCD depth pairing influences portal dosimetry performance, particularly for flattened beams, underscoring the importance of depth-aware configuration in QA protocols.

## INTRODUCTION

Portal dosimetry has become an essential component of patient-specific quality assurance (QA) in modern radiation therapy, particularly for techniques such as intensity-modulated radiation therapy (IMRT) and volumetric modulated arc therapy (VMAT), where precise verification of complex fluence distributions is critical [1–5]. Among available QA methods, the portal dosimetry system offers a fast and non-invasive approach to assess fluence agreement between planned and delivered treatments. In this workflow, predicted images generated using the portal dosimetry image prediction (PDIP) algorithm within the treatment planning system (TPS) are compared with portal images acquired using an electronic portal imaging device (EPID).

A key element of this process is the incorporation of off-center diagonal (OCD) profiles—beam profiles measured at various off-axis depths—into both the TPS and the EPID system. In the TPS, these OCD profiles are used within the PDIP algorithm to generate predicted fluence images. In the EPID system, the same profiles are registered to correct the measured fluence. However, despite their central role in the accuracy of portal dosimetry, current guidelines provide limited direction on how the selection and pairing of OCD profile depths affect verification accuracy [6–8].

This consideration becomes particularly important in large field sizes and high-energy photon beams, where off-axis dose characteristics vary significantly with depth. Although QA procedures and dosimetric validation techniques for portal dosimetry are well established [7–10], most previous studies have either used fixed OCD profile settings or focused on detector-specific factors [11, 12]. For example, Kerns *et al*. [13] investigated inter-institutional consistency in beam modeling parameters, highlighting the importance of accurate profile configuration for reproducibility in patient-specific QA. However, their study did not evaluate how different OCD profile depth settings between the TPS and EPID systems may influence verification accuracy.

While portal dosimetry is widely implemented in clinical practice, the impact of such depth configurations has not been systematically assessed. Hobson and Davies reported that flattened (FF) beams are generally more sensitive to modeling conditions than flattening filter-free (FFF) beams, but their analysis did not consider the effect of OCD depth pairing between prediction and measurement systems [14]. Similarly, Pardo *et al*. investigated the robustness of FFF beams in portal dosimetry but did not examine how OCD depth pairing between TPS and EPID systems might influence verification outcomes [15].

In this study, 25 combinations of OCD profile depths (5 in TPS × 5 in EPID) were systematically evaluated across multiple photon energies and square field sizes. Here, ‘pairing’ refers to the combination of one TPS OCD depth with one EPID OCD depth. Rather than suggesting a general energy dependence, our findings demonstrate that optimal gamma pass rates depend on specific beam configurations defined by energy and field size. This indicates that no single OCD profile depth is universally optimal; instead, the best verification accuracy is achieved through configuration-specific OCD depth pairing between TPS and EPID systems. To our knowledge, this is the first study to provide empirical evidence that OCD profile pairing significantly influences portal dosimetry outcomes, with direct implications for improving clinical QA protocols.

## MATERIALS AND METHODS

### Predicted fluence model in PDIP

The PDIP algorithm calculates predicted fluence images based on beam modeling parameters such as beam profiles, output factors and scatter corrections. According to the vendor’s documentation [16], the predicted portal dose image *P*(*x*,*y*) is calculated as:


(1) \begin{equation*}P\left(x,y\right)={f}^{\prime}\left(x,y\right)\cdotp k\cdotp \left({\mathrm{SAD}}^2/{\mathrm{SDD}}^2\right)\cdotp \left[\mathrm{OF}\left( fs_{x}, fs_{y}\right)/\mathrm{PSF}\left( fs_{x}, fs_{y}\right)\right]\end{equation*}

where *f ′*(*x,y*) is the input fluence modulated by the beam intensity profile. *k* is the dose kernel of the portal imager. SAD is the source-to-axis distance. SDD is the source-to-detector distance. OF(*fs_x_*, *fs_y_*) is the output factor for field size *fs_x_ × fs_y_.* PSF(*fs_x_*, *fs_y_*) is the phantom scatter factor for the same field size.

The modulated fluence *f ′*(*x,y*) in Equation (1) is defined as:


(2) \begin{equation*}{f}^{\prime}\left(x,y\right)=f\left(x,y\right)\cdotp v\left(r_{{x},{y}}\right)\end{equation*}

where *f*(*x*,*y*) is the uncorrected fluence from the treatment plan and *v*(*r_x,y_*) is the radial intensity modulation function based on off-axis distance.

As shown in Equation (2), the beam profile function *v*(*r_x,y_*), although not explicitly defined as a function of depth, is empirically known to vary with measurement depth. In clinical implementation, it is registered independently in both the TPS and the EPID. This study investigates how different depth settings for *v*(*r_x,y_*) in TPS and EPID affect fluence prediction accuracy and gamma pass rates. While the full PDIP algorithm remains proprietary, this formulation reflects key components validated in the Varian Reference Guide [16].

### OCD profile acquisition

OCD profiles were acquired using a BEAMSCAN water phantom system (PTW, Germany) with a Semiflex 3D ionization chamber (Type 31 021, PTW). Measurements were conducted under reference conditions (field size: 40 × 40 cm^2^, SSD: 100 cm). Lateral profiles were acquired along the diagonal axis through the beam center to characterize symmetric off-axis behavior.

Profiles were measured at five depths in water: the reference depth (defined below), 5, 10, 20 and 30 cm. For each photon energy (6X, 10X, 6FFF, 10FFF), static fields at gantry angle 0° were used. Profile data were normalized to the central axis and exported in the required format for registration in both the TPS and EPID systems.

The reference depth d*r*, corresponding to the depth of maximum dose, was defined per beam energy: 1.5 cm (6X), 2.3 cm (10X), 1.3 cm (6FFF) and 2.2 cm (10FFF). These values were used as nominal reference profiles for each beam.

### Treatment planning and fluence prediction

In the PDIP algorithm used by Eclipse for portal dose prediction, one OCD profile must be registered. To investigate potential depth dependence, five different OCD profiles (d*r*, 5, 10, 20 and 30 cm) were prepared and separately registered, resulting in five distinct PDIP models per energy.

On the EPID side, measured dose images also require registration of one OCD profile for correction; therefore, the same set of five OCD profiles was registered. In this study, ‘pairing’ is defined as the combination of the OCD depth used for TPS beam modeling with the OCD depth used for EPID correction. This resulted in 25 unique TPS–EPID pairings per energy. For example, a TPS model constructed with the 10 cm OCD (D10) could be evaluated against EPID measurements corrected with the 5 cm OCD (E05), 20 cm OCD (E20) or the reference depth (*E*d*r*).

Treatment plans consisted of square fields in seven sizes: 5 × 5, 7 × 7, 10 × 10, 12 × 12, 15 × 15, 20 × 20 and 30 × 30 cm^2^. For each energy, five PDIP models were configured using different OCD profile depths in the TPS (*D*d*r*, D05, D10, D20, D30), resulting in 35 plans per energy (5 depths × 7 fields).

Each plan was measured under five EPID configurations, with beam profiles registered at the same five depths (*E*d*r*, E05, E10, E20, E30), yielding 175 plan–measurement combinations per energy. A schematic of the full measurement workflow—including TPS/EPID depth pairings—is provided in Supplementary Fig. S1.

### EPID fluence measurements

Portal images were acquired using an aS1200 EPID mounted on a TrueBeam linear accelerator (Varian Medical Systems, Palo Alto, CA). The EPID features a 1280 × 1280 pixel matrix with a 0.34 mm pixel pitch, covering an approximate area of 43 × 43 cm^2^.

Five OCD profiles—measured at d*r*, 5, 10, 20 and 30 cm depths—were registered in the EPID system for use in fluence correction during portal image acquisition. For each EPID configuration, one OCD profile was applied, and all 35 treatment plans (created using 5 TPS OCD depths × 7 field sizes) were measured. This process was repeated for all five EPID profiles, resulting in 175 depth pairing combinations per energy. All images were acquired at a source-to-imager distance of 100 cm, following the standard clinical calibration geometry recommended for the Varian aS1200 EPID. Weekly EPID calibration was performed using the vendor-supplied protocol, and output constancy was verified using IsoCal and routine QA.

### Gamma analysis

Gamma analysis was performed using Portal Dosimetry software (version 16.1, Varian). For each energy (4 photon energies), 35 treatment plans were generated from five PDIP models and seven square field sizes. Each plan was evaluated against five EPID images acquired with different OCD profiles, resulting in a total of 700 plan–measurement comparisons (5 PDIP depths × 7 fields × 5 EPID depths × 4 energies).

The gamma evaluation employed global 3%/3 mm criteria with a 10% dose threshold, in accordance with AAPM TG-218 recommendations [10]. This tolerance level is also consistent with typical IMRT QA including portal dosimetry, as summarized in AAPM TG-307 [17]. To further investigate the sensitivity of the analysis, especially for FFF beams that showed near-100% pass rates under 3%/3 mm, supplementary evaluation was also performed using a stricter 2%/2 mm criterion, as suggested in AAPM MPPG 5.a [18]. The results of this additional analysis are presented in the Supplementary Materials (Supplementary Figs S2 and S3). The gamma pass rate—defined as the percentage of pixels with γ ≤ 1.0—served as the primary metric for analysis.

### Data analysis strategy

For each photon energy and field size, gamma pass rates were summarized using the mean, standard deviation (SD), maximum and minimum values. Heatmaps were generated to visualize depth-dependent trends.

For the 6X and 10X beams, a two-way analysis of variance (ANOVA) was performed to assess the effects of TPS OCD profile depth, EPID OCD profile depth and their interaction. *Post hoc* comparisons were conducted using Tukey’s honestly significant difference (HSD) test. All statistical analyses were performed using SPSS (version 28.0, IBM Corp.), with statistical significance set at *P* < 0.05. Effect sizes were reported using eta squared (*η^2^*).

Due to the limited variability in gamma pass rates for the 6FFF and 10FFF beams (all >95%, SD < 1%), these datasets were excluded from the ANOVA and are instead presented in Supplementary Figures.

## RESULTS

### Overview of gamma pass rates

Gamma pass rates varied across photon energies and field sizes, depending on the combination of OCD profile depths used in the PDIP model and EPID image correction. For the 6X beam, substantial variation was observed. The highest gamma pass rate (100.0%) was achieved when the EPID OCD depth was set to 30 cm and the PDIP OCD depth was either 20 or 30 cm (D20_E30 or D30_E30). In contrast, the lowest pass rate (71.5%) was observed under a mismatched configuration (D30_Edr).

For the 10X beam, the maximum pass rate (100.0%) occurred for the D20_E30 pairing, while the minimum (58.5%) was again recorded for D30_Edr.

**Fig. 1:**
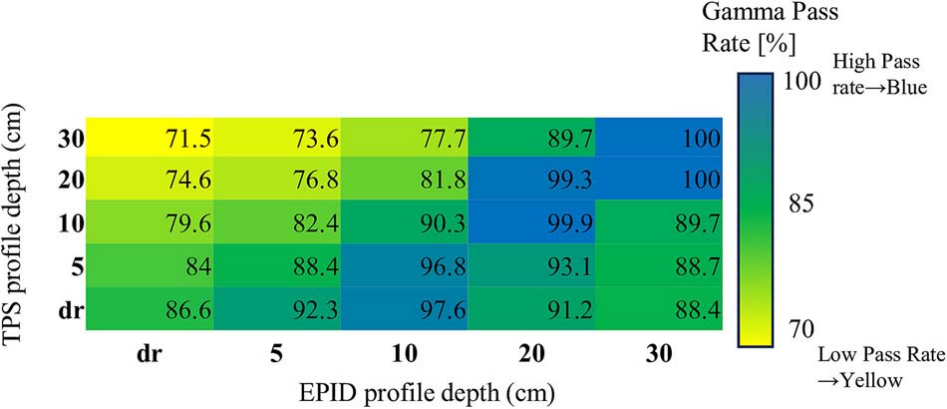
Heatmap of average gamma pass rates for the 6X beam, showing 25 combinations of TPS and EPID OCD profile depths. Each cell represents the mean gamma pass rate (%) across seven square field sizes, calculated using the 3%/3 mm gamma criterion with a 10% dose threshold. The accompanying scale bar indicates pass rates ranging from 70% to 100%, with higher values representing greater agreement between TPS and EPID depth pairings. The diagonal line corresponds to matched depth settings. TPS = treatment planning system, EPID = electronic portal imaging device, OCD = off-center diagonal.

**Fig. 2:**
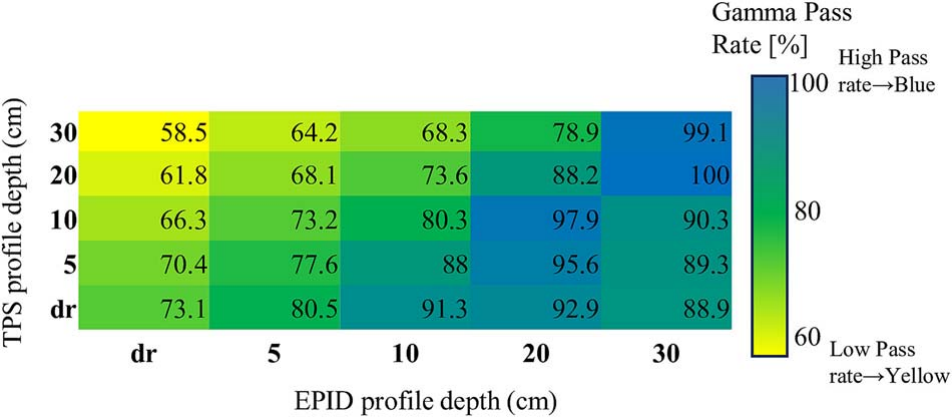
Heatmap of average gamma pass rates for the 10X beam, showing 25 combinations of TPS and EPID OCD profile depths. Each cell represents the mean gamma pass rate (%) across seven square field sizes, calculated using the 3%/3 mm gamma criterion with a 10% dose threshold. The scale bar indicates pass rates ranging from 60% to 100%, with higher values representing stronger agreement between TPS and EPID depth pairings. The diagonal line represents matched depth settings.

Figures 1 and 2 show heatmaps of the mean gamma pass rate distributions for the 6X and 10X beams, respectively. Summary results from the two-way ANOVA examining the effects of PDIP and EPID depth settings are presented in Supplementary Tables S1 and S2.

To further assess the stability and reproducibility of gamma pass rates across different field sizes, we calculated the standard deviation (SD) for each TPS–EPID OCD depth pairing. The results are visualized as heatmaps in Supplementary Figs S4 (6X) and S5 (10X). These maps highlight that depth-mismatched configurations generally exhibited greater variability, with SD values exceeding 25% in some combinations. In contrast, depth-aligned pairs such as D20_E20 and D30_E30 showed consistently low SD values (<5%), supporting their robustness and consistency in clinical verification.

### Statistical analysis by ANOVA

A two-way ANOVA was performed to evaluate the effects of PDIP OCD depth, EPID OCD depth and their interaction on gamma pass rates for the 6X and 10X beams. For both energies, the main effects of PDIP and EPID OCD depths were statistically significant (*P* < 0.001; see Supplementary Tables S1 and S2). The interaction effect was significant for the 10X beam (*P* = 0.024), but not for the 6X beam (*P* = 0.073).

*Post hoc* comparisons using Tukey’s HSD test identified specific OCD depth pairings associated with significantly higher or lower gamma pass rates. For the 6X beam, depth-aligned configurations such as D30_E30 and D20_E30 yielded significantly higher pass rates than mismatched pairings like D30_Edr (*P* < 0.01). A similar trend was observed for the 10X beam, where D20_E30 outperformed several mismatched configurations, including D30_Edr, with adjusted *P*-values < 0.05.

Detailed pairwise comparisons and associated *P*-values are presented in Supplementary Table S3.

These findings support the hypothesis that proper alignment between PDIP and EPID OCD profile depths enhances agreement between predicted and measured portal dose images. Conversely, gamma pass rates may be substantially degraded under mismatched configurations, highlighting the clinical importance of depth pairing.

### Gamma pass rates for FFF beams

For the 6FFF and 10FFF beams, gamma pass rates consistently exceeded 95% across all PDIP–EPID OCD depth pairings and field sizes. Variability was minimal, with standard deviations below 1.0% in all cases. Accordingly, no additional statistical analysis was performed for these energies.

**Fig. 3:**
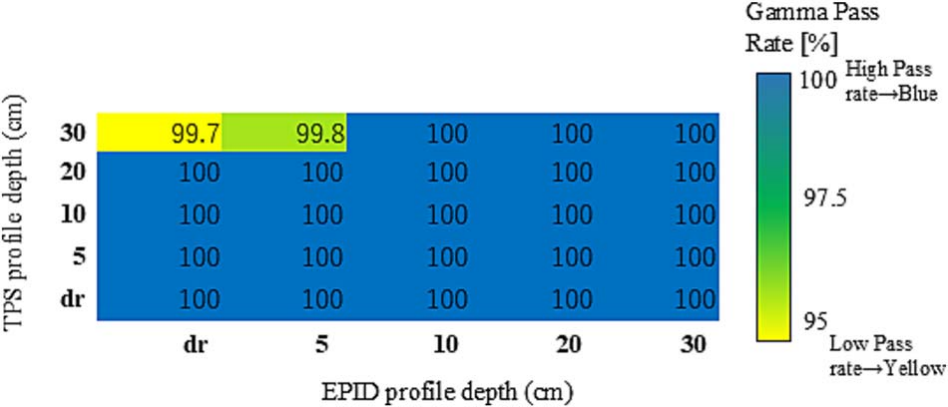
Heatmap of average gamma pass rates for the 6FFF photon beam, calculated across seven square field sizes using the 3%/3 mm gamma criteria with a 10% dose threshold. Each cell represents the mean gamma pass rate (%) for a specific combination of TPS and EPID profile depths. The color scale is set from 95% to 100% to enhance the visualization of subtle variations in agreement.

Mean gamma pass rate distributions are presented as heatmaps in Figs 3 (6FFF) and 4 (10FFF). To further examine the sensitivity of the evaluation, supplementary analysis with the stricter 2%/2 mm criterion was performed. For 6FFF, gamma pass rates dropped to the 70% range when the TPS OCD depth was set to 30 cm in combination with shallow EPID OCD depths (e.g. D30_Edr), while all other combinations maintained pass rates above 95%. For 10FFF, most pairings also remained above 95%, but reduced pass rates were observed when the TPS OCD depth was set to 20 or 30 cm, in some cases down to the 70% range. Importantly, the locations of reduced pass rates in the 2%/2 mm analysis closely matched those observed in the 3%/3 mm analysis, indicating that the depth dependence is a robust effect and not substantially influenced by the chosen gamma criterion. The distributions of gamma pass rates from this supplementary analysis are presented as heatmaps in Supplementary Figs S2 (6FFF) and S3 (10FFF). These results confirm the robustness of fluence verification for FFF beams, which appear largely insensitive to variations in OCD depth pairing. This suggests that the PDIP algorithm provides inherently stable modeling performance for FFF beams, regardless of depth configuration.

**Fig. 4:**
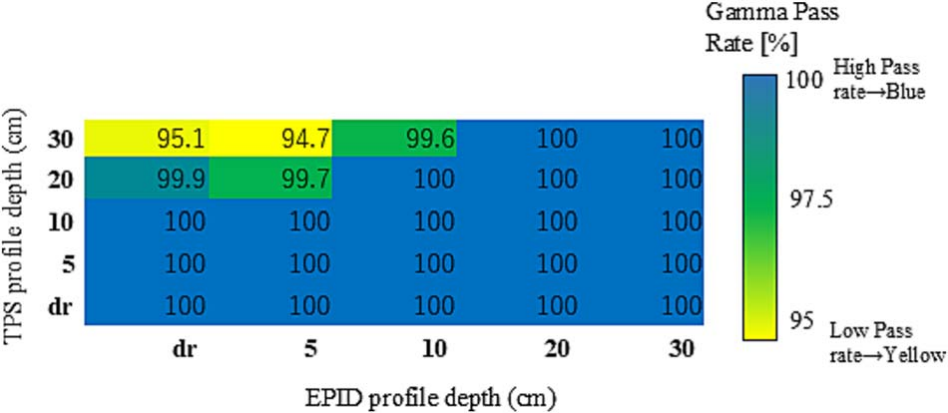
Heatmap of average gamma pass rates for the 10FFF photon beam, calculated across seven square field sizes using the 3%/3 mm gamma criteria with a 10% dose threshold. Each cell represents the mean gamma pass rate (%) for a specific combination of TPS and EPID profile depths. The color scale is set from 95% to 100% to enhance the visualization of subtle variations in agreement.

## DISCUSSION

This study demonstrated that gamma agreement between predicted and measured portal dose images is influenced by the pairing of OCD profile depths configured in the TPS and the EPID, with the effect reaching statistical significance in the 10X beam and showing a similar trend in the 6X beam. The 6X and 10X beams were particularly sensitive to OCD depth pairing, with gamma pass rates varying substantially across different TPS–EPID configurations. In contrast, the 6FFF and 10FFF beams consistently achieved high pass rates across all pairings, indicating greater robustness to depth mismatch. These findings provide new evidence of the PDIP algorithm’s sensitivity to profile configuration and underscore the importance of depth alignment in patient-specific QA workflows.

In the 6X and 10X beams, gamma pass rates were notably reduced when the OCD depth in the PDIP model did not match that registered in the EPID system. Even modest mismatches resulted in significant degradation in agreement. While previous studies (e.g. Hobson and Davies [14], Pardo *et al*. [15]) examined aspects of PDIP modeling and verification, none systematically evaluated the impact of OCD profile depth pairing. The present study provides quantitative evidence that such alignment plays a meaningful role in fluence verification accuracy—particularly in the 10× beam where interaction effects were statistically significant—and should be carefully considered during PDIP commissioning and QA protocol development.

These results have direct implications for clinical QA in IMRT and VMAT. For the 6X and 10X beams, suboptimal depth pairings can degrade gamma pass rates, potentially triggering unnecessary plan reviews or treatment delays. Therefore, OCD depth alignment should be considered a key configuration parameter during both initial model setup and ongoing QA maintenance. Conversely, the observed insensitivity of FFF beams to depth pairing suggests that QA workflows for FFF deliveries may allow for greater flexibility without compromising fluence verification accuracy.

For the 6FFF and 10FFF beams, average gamma pass rates remained consistently above 95% across most profile pairings under the 3%/3 mm criterion, as shown in Figs 3 and 4. While minor differences were observed between TPS and EPID configurations, all mean values exceeded clinical acceptance thresholds.

The 3%/3 mm criterion was adopted in this study because it represents the most widely used tolerance in clinical IMRT QA and is explicitly recommended in AAPM TG-218 [10]. Furthermore, AAPM TG-307 [17] notes that 3%/3 mm is the predominant benchmark in portal dosimetry practice. To further examine the robustness of our findings, we also performed a supplementary analysis using a stricter 2%/2 mm criterion. For 6FFF, gamma pass rates decreased to the 70% range when the TPS OCD depth was set to 30 cm and paired with shallow EPID OCD depths (e.g. D30_Edr), while all other pairings consistently remained above 95%. For 10FFF, most pairings also remained above 95%, but reduced pass rates were observed when the TPS OCD depth was set to 20 or 30 cm, again with some cases falling into the 70% range. Importantly, the locations of reduced pass rates in the 2%/2 mm analysis closely matched those already observed under the 3%/3 mm criterion. This indicates that the depth dependence is a robust phenomenon rather than an artifact of the tolerance level. Accordingly, the 2%/2 mm analysis should be considered a useful supplementary evaluation, providing additional sensitivity while the 3%/3 mm threshold remains the clinical benchmark for portal dosimetry.

These results suggest that FFF beams exhibit reduced sensitivity to OCD profile depth pairing, although statistical fluctuations may still occur depending on the specific setup.

Collectively, across both FF and FFF beams, the observed depth dependence can be explained by differences in the shape of OCD profiles measured at varying depths, which directly affect the accuracy of PDIP fluence modeling.

This work supports the refinement of current QA protocols by incorporating depth alignment as a validation step—particularly in institutions employing the 6X and 10X beams. Furthermore, the findings highlight the need for standardized modeling practices, as increasingly emphasized in recent recommendations such as AAPM Task Group 307 [17]. As QA systems evolve toward automation and machine learning-assisted processes, accurate beam modeling—especially of off-axis fluence characteristics—will remain essential to ensuring reliable downstream verification.

Several limitations should be acknowledged. First, all measurements and modeling were conducted using a single TPS–linac system within a single institution. Although depth-dependent effects were clearly demonstrated, the generalizability of optimal depth pairings to other platforms or vendors remains uncertain. Second, statistical analyses were limited to the 6X and 10X beams due to the minimal variability observed in the FFF results. Future studies may incorporate higher-resolution metrics or alternative comparison methods to detect subtler trends in FFF beam behavior.

Finally, although this study focused on OCD profile depth pairing, other model parameters—such as effective source size, off-axis softening and multileaf collimator modeling—were not examined. Future research should incorporate multi-institutional datasets, broader modeling parameters and alternative verification approaches to validate and extend the clinical relevance of depth alignment in PDIP-based QA.

Based on the findings, we identified specific OCD depth pairings that consistently achieved gamma pass rates exceeding 98% across all field sizes under 3%/3 mm criteria. These configurations may serve as institution-specific reference pairings for commissioning of the Portal Dosimetry system and for QA planning. Although derived from data at a single site, this reference guide may offer a practical starting point for other facilities seeking to establish their own OCD pairing strategies. Broader validation across institutions is warranted to develop generalized decision frameworks.

## CONCLUSION

This study demonstrated that gamma pass rates in portal dosimetry are influenced by the pairing of OCD profile depths configured in the TPS and EPID, particularly for the 6X and 10X beams. Fluence verification was most consistently achieved when OCD depths were appropriately aligned between the PDIP model and EPID configuration. In contrast, FFF beams exhibited reduced sensitivity to such variations, indicating greater robustness in clinical QA workflows.

These findings highlight the importance of OCD depth alignment during portal dosimetry commissioning and QA protocol development, particularly for FF beam deliveries. While this analysis was conducted at a single institution, the observed depth-dependent effects emphasize the need for standardized modeling practices and multi-institutional validation. Incorporating OCD pairing considerations into routine QA processes may enhance the consistency and reliability of portal dosimetry in modern radiation therapy.

## Supplementary Material

Supplementary_Figures_rraf071

Supplementary_Tables_rraf071
