# Supplementary material for: Impact of off-center diagonal profile depth pairing on gamma pass rates in portal dosimetry
Source: J Radiat Res. 2025 Nov 24;67(1):78–83. doi: 10.1093/jrr/rraf071 (PMC12856035; doi:10.1093/jrr/rraf071)
Supplement: Supplementary_Figures_rraf071 [file supplementary_figures_rraf071.pdf]

Supplementary Figures

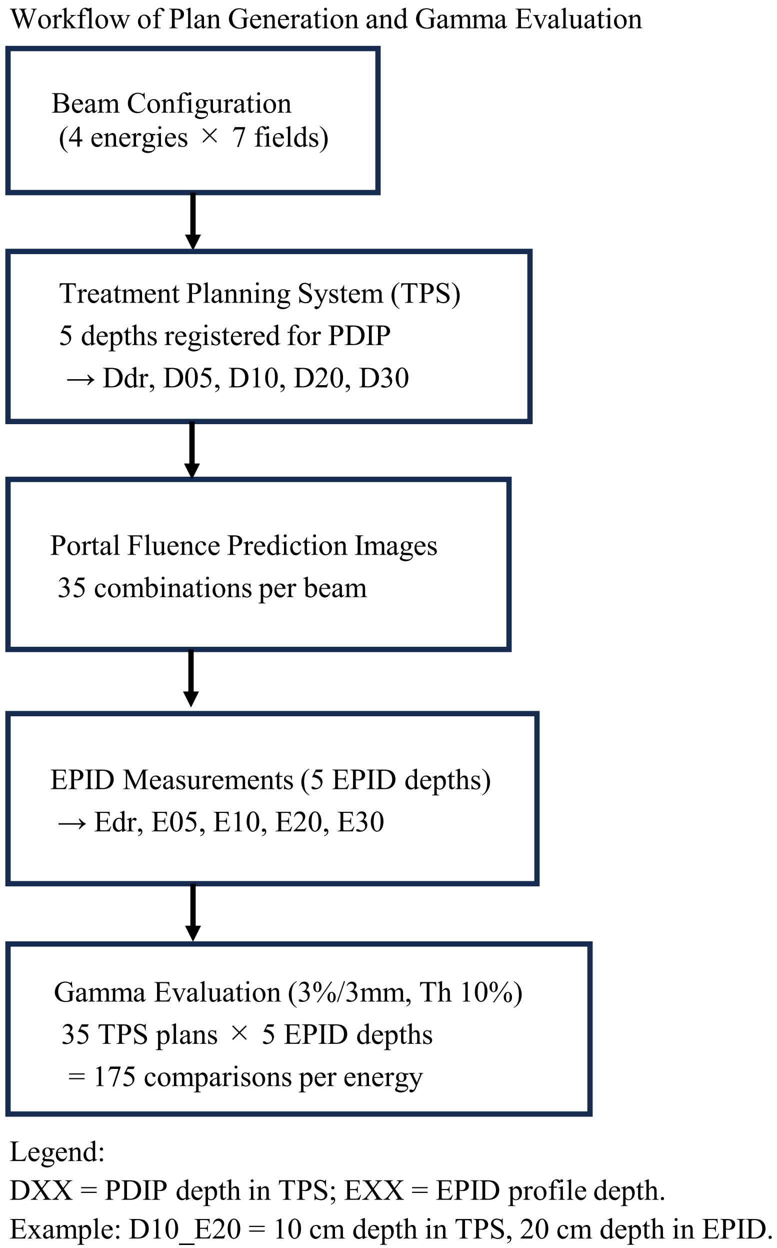

Supplementary Figure_S1. Workflow illustrating the creation of treatment plans with various PDIP profile depth settings and their pairing with EPID acquisitions at corresponding depths. Each plan was labeled using the convention DXX_EXX (e.g., D10_E20 indicates a 10 cm depth in the TPS and a 20 cm depth in the EPID). For each photon energy, a total of 175 plan–acquisition combinations were evaluated using gamma analysis (3%/3 mm, 10% threshold).

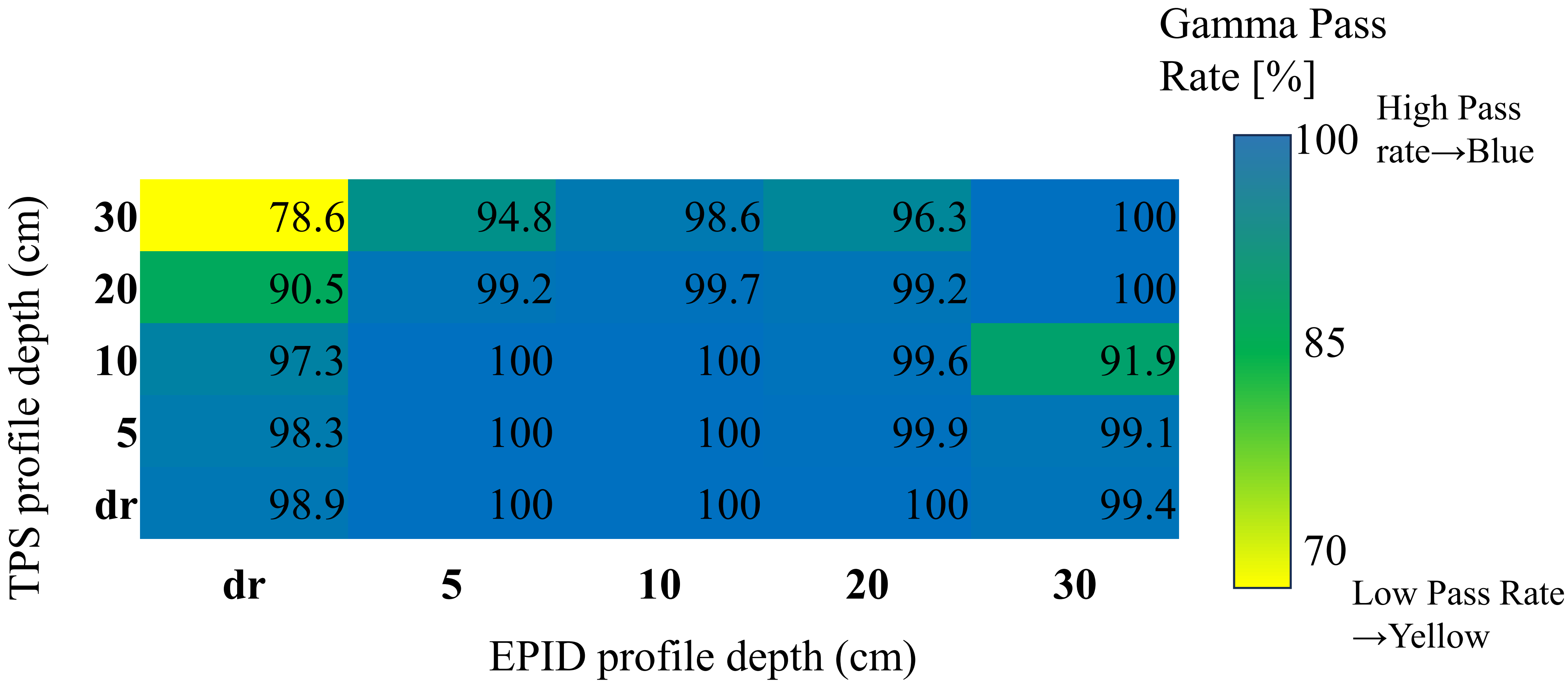

Supplementary Figure_S2. Heatmap of average gamma pass rates for the 6FFF photon beam, calculated across seven square field sizes using the 2%/2 mm gamma criteria with a 10% dose threshold. Each cell represents the mean gamma pass rate (%) for a specific combination of TPS and EPID profile depths. The color scale is set from 70% to 100% to enhance the visualization of subtle variations in agreement

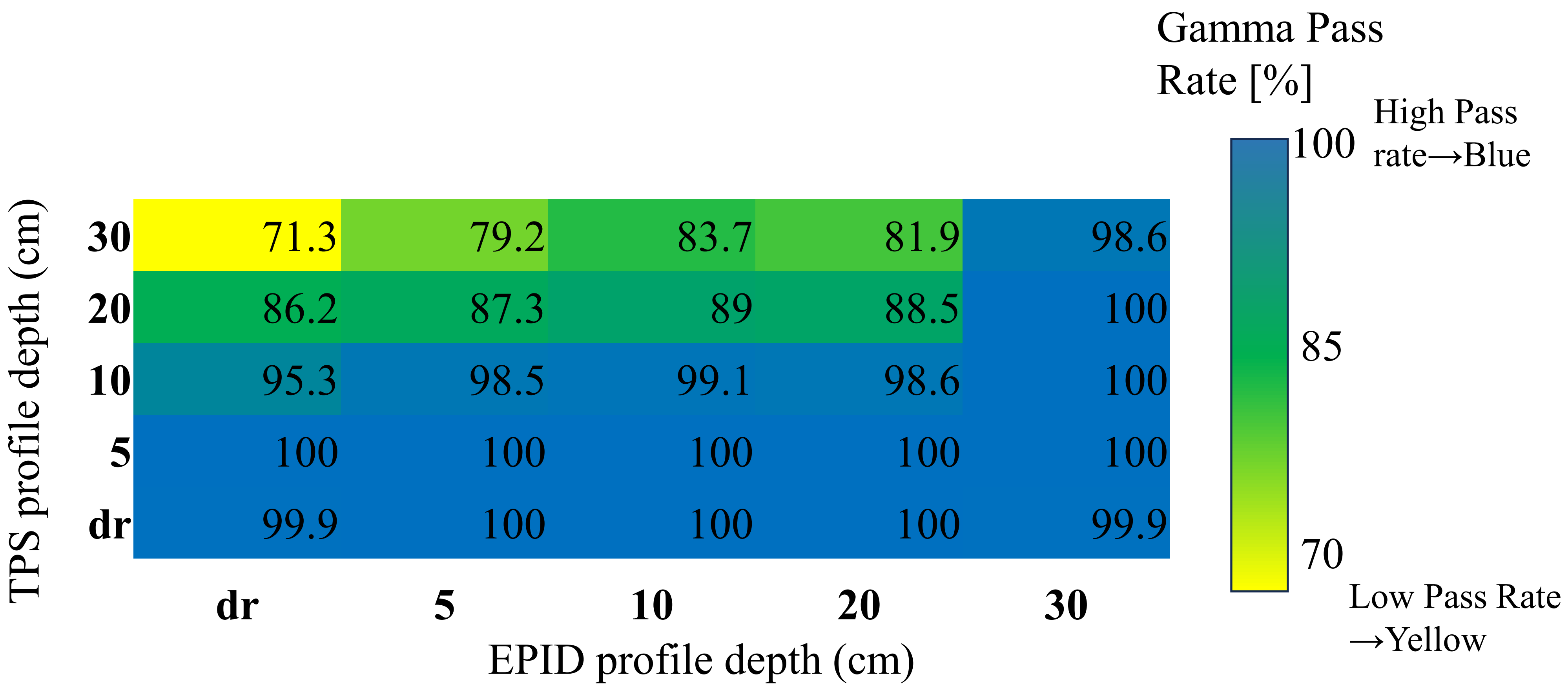

Supplementary Figure_S3. Heatmap of average gamma pass rates for the 10FFF photon beam, calculated across seven square field sizes using the 2%/2 mm gamma criteria with a 10% dose threshold. Each cell represents the mean gamma pass rate (%) for a specific combination of TPS and EPID profile depths. The color scale is set from 70% to 100% to enhance the visualization of subtle variations in agreement.

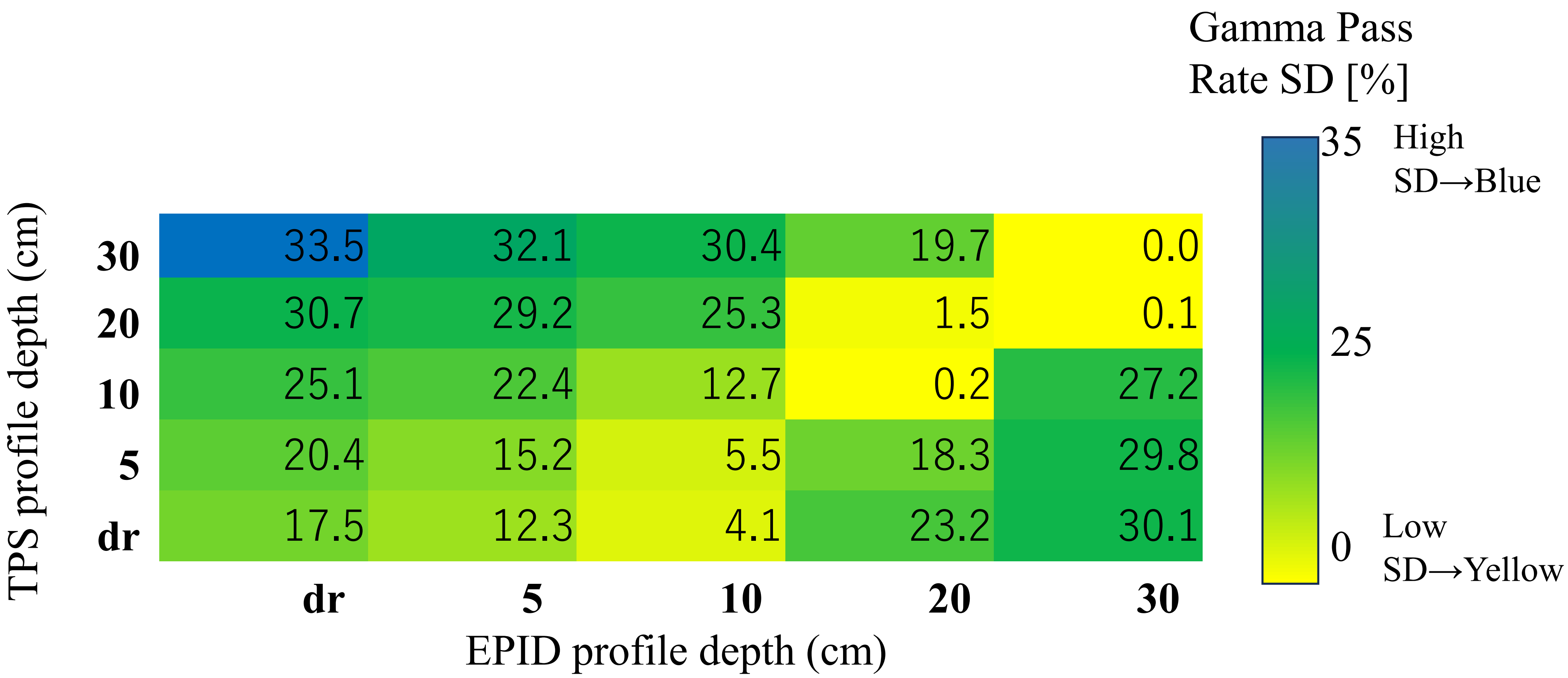

Supplementary Figure_S4. Heatmap of the standard deviation (SD) of gamma pass rates for the 6 MV photon beam across seven square field sizes. Each cell represents the SD of the gamma pass rate (%) for a specific combination of TPS and EPID profile depths, based on gamma analysis using the 3%/3 mm criteria with a 10% dose threshold. The color bar ranges from low (yellow) to high (blue) SD values, indicating the degree of variability in agreement between TPS and EPID depth combinations.

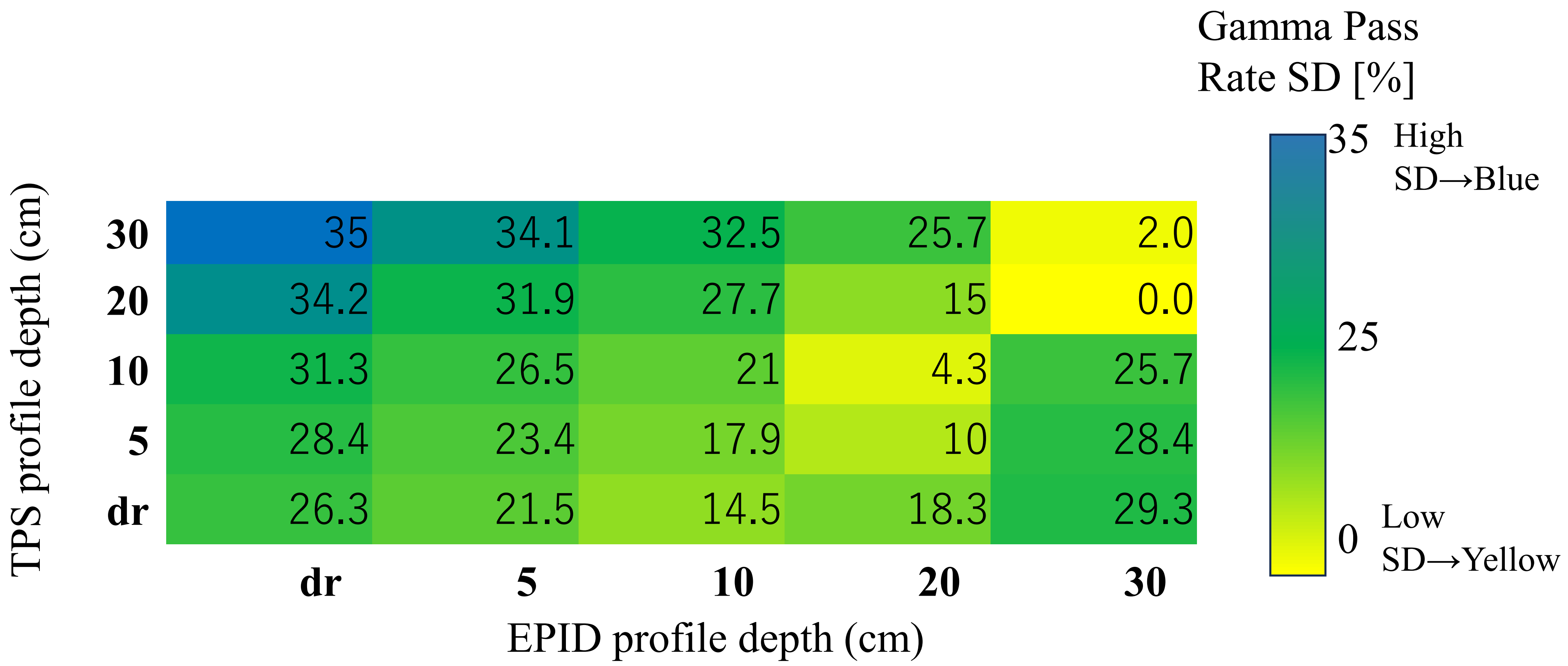

Supplementary Figure_S5. Heatmap of the standard deviation (SD) of gamma pass rates for the 10 MV photon beam across seven square field sizes. Each cell represents the SD of the gamma pass rate (%) for a specific combination of TPS and EPID profile depths, based on gamma analysis using the 3%/3 mm criteria with a 10% dose threshold. The color bar ranges from low (yellow) to high (blue) SD values, indicating the degree of variability in agreement between TPS and EPID depth combinations.
