# Supplementary material for: Impact of off-center diagonal profile depth pairing on gamma pass rates in portal dosimetry
Source: J Radiat Res. 2025 Nov 24;67(1):78–83. doi: 10.1093/jrr/rraf071 (PMC12856035; doi:10.1093/jrr/rraf071)
Supplement: Supplementary_Tables_rraf071 [file supplementary_tables_rraf071.pdf]

Supplementary Tables

Supplementary Table_S1. Summary of two-way ANOVA Analysis of gamma pass rates (%) for the 6X beam

Factor	df	SS	MS	F	p-value	η^2
PDIP depth	4	225.97	56.49	19.54	**<0.001**	0.275
EPID depth	4	265.49	66.37	22.96	**<0.001**	0.323
PDIP × EPID interaction	16	74.23	4.64	1.61	0.073	0.090
Error	150	433.63	2.89			
Total	174	999.32				

Abbreviations: df = degrees of freedom; SS = sum of squares; MS = mean square; F = F-statistic; η^2 = eta squared (effect size).

Supplementary Table_S2. Summary of two-way ANOVA Analysis of gamma pass rates (%) for the 10X beam

**Factor**	**df**	**SS**	**MS**	**F**	**p-value**	**η^2**
PDIP depth	4	381.25	95.31	41.21	**<0.001**	0.431
EPID depth	4	155.48	38.87	16.8	**<0.001**	0.176
PDIP × EPID interaction	16	70.45	4.4	1.9	0.024	0.08
Error	150	346.9	2.31			
Total	174	953.57				

Abbreviations: df = degrees of freedom; SS = sum of squares; MS = mean square; F = F-statistic; η^2 = eta squared (effect size).

Supplementary Table_S3. Pairwise comparisons using Tukey's HSD test for γ-pass rates across PDIP–EPID depth combinations (6X and 10X beams)

Energy	PDIP Depth	EPID Depth	Mean Difference (%)	Adjusted p-value	Significant ($p < 0.05$)
6X	30 cm	dr	–28.5	< 0.01	Yes
6X	30 cm	10 cm	–15.2	< 0.01	Yes
6X	30 cm	20 cm	–9.7	< 0.01	Yes
10X	30 cm	dr	–41.5	< 0.01	Yes
10X	30 cm	10 cm	–23.6	< 0.01	Yes
10X	20 cm	30 cm	7.2	< 0.05	Yes

Note: Only depth combinations with statistically significant differences (adjusted $p < 0.05$) are shown.

Supplementary Table_S3. Tukey's HSD pairwise comparisons of gamma pass rates for selected PDIP–EPID profile depth combinations using 6 MV and 10 MV photon beams. The table presents mean differences (%) and adjusted p-values for significantly different combinations identified in the ANOVA. Results are averaged over seven square field sizes.
